# Biochemical and physiological flexibility accompanies reduced cellulose biosynthesis in Brachypodium *cesa1*^*S830N*^

**DOI:** 10.1093/aobpla/plz041

**Published:** 2019-07-13

**Authors:** Chad Brabham, Abhishek Singh, Jozsef Stork, Ying Rong, Indrajit Kumar, Kazuhiro Kikuchi, Yaroslava G Yingling, Thomas P Brutnell, Jocelyn K C Rose, Seth Debolt

**Affiliations:** 1 Department of Horticulture, University of Kentucky, Lexington, KY, USA; 2 Department of Materials Science and Engineering, North Carolina State University, Raleigh, NC, USA; 3 Donald Danforth Plant Science Center, St. Louis, MO, USA; 4 KWS Gateway Research Center, St. Louis, MO, USA; 5 Syngenta Japan K.K., Chuo-ku, Tokyo, Japan; 6 Plant Biology Section, School of Integrative Plant Science, Cornell University, Ithaca, NY, USA

**Keywords:** Biomechanics, cellular expansion, cellulose, CESA, class-specific region, molecular dynamics

## Abstract

Here, we present a study into the mechanisms of primary cell wall cellulose formation in grasses, using the model cereal grass *Brachypodium distachyon*. The exon found adjacent to the Bd*CESA1* glycosyltransferase QXXRW motif was targeted using Targeting Induced Local Lesions in Genomes (TILLING) and sequencing candidate amplicons in multiple parallel reactions (SCAMPRing) leading to the identification of the Bd*cesa1*^*S830N*^ allele. Plants carrying this missense mutation exhibited a significant reduction in crystalline cellulose content in tissues that rely on the primary cell wall for biomechanical support. However, *Bdcesa1*^*S830N*^ plants failed to exhibit the predicted reduction in plant height. In a mechanism unavailable to eudicotyledons, *B. distachyon* plants homozygous for the *Bdcesa1*^*S830N*^ allele appear to overcome the loss of internode expansion anatomically by increasing the number of nodes along the stem. Stem biomechanics were resultantly compromised in Bd*cesa1*^*S830N*^. The *Bdcesa1*^*S830N*^ missense mutation did not interfere with *BdCESA1* gene expression. However, molecular dynamic simulations of the CELLULOSE SYNTHASE A (CESA) structure with modelled membrane interactions illustrated that Bd*cesa1*^*S830N*^ exhibited structural changes in the translated gene product responsible for reduced cellulose biosynthesis. Molecular dynamic simulations showed that substituting S830N resulted in a stabilizing shift in the flexibility of the class specific region arm of the core catalytic domain of CESA, revealing the importance of this motion to protein function.

## Teaser

Despite the majority of terrestrial biomass being produced by grasses, few studies have investigated the process of cellulose biosynthesis in monocotyledonous species. It is worth noting that based on phylogenetic studies, cellulose biosynthetic genes appear quite conserved across monocotyledons and dicotyledonous plants. There are however some important differences. In this study *Brachypodium distachyon* was used to isolate a S830N amino acid substitution in CELLULOSE SYNTHASE (CESA)1. Whole-plant physiological studies were used to learn about the impact of reduced cellulose biosynthesis on grasses. Furthermore, we used modelling to make predictions about how subtle changes in the amino acid structure influenced CESA1 function.

## Introduction

Recent advances in molecular techniques have facilitated significant progress in the field of plant functional genomics. However, most such studies focus on model organisms, with the eudicotyledonous *Arabidopsis thaliana* (arabidopsis), leading the way. Indeed, great strides have been made in developing a mechanistic understanding of cellulose biosynthesis by studies of arabidopsis including the determination of genes involved in the heterotrimeric cellulose synthase complex (CSC) (Arioli *et al.* 1998; Schieble *et al.* 2001; Desprez *et al.* 2002, 2007; Kurek *et al.* 2002; Taylor *et al.* 2003), structure-function relationships of CSC components (Harris *et al.* 2012; Sethaphong *et al.* 2013; Slabaugh *et al.* 2014) and an active field of study into CSC accessory proteins (Endler *et al.* 2015; Lei *et al.* 2015). Furthermore, X-ray crystallography has resolved the bacterial cellulose synthase protein structure (Morgan *et al.* 2013), which is distinct from plants in many ways. In plants, the homomeric structure of a *Gossipium hirsutum* CELLULOSE SYNTHASE A (CESA) has recently been modelled (Nixon *et al.* 2016) and assembled *in vitro* (Purushotham *et al.* 2016), revealing numerous unanswered structural questions.

The family Poaceae is the most economically important group of plants and includes crops such as cereals, forage grasses, biofuel feedstocks and a variety of weed species. The temperate, C3 annual grass, *Brachypodium distachyon* (*B. distachyon* or brachypodium) (Poales: Poaceae) has emerged as a model grass for molecular genetic studies (Draper *et al.* 2001; Vogel *et al.* 2010). Moreover, several studies have advanced brachypodium as a genetic model for grass cell wall development (Christensen *et al.* 2010), cereal–pathogen interactions (Fitzgerald *et al.* 2015) and grain development (Hands and Drea 2012).

In eudicots, the primary cell wall comprises approximately a 1:1:1 ratio of cellulose, hemicellulose (mainly xyloglucans) and an assortment of pectic polysaccharides. Cellulose is the major structural component and the biosynthetic machinery responsible for cellulose biosynthesis has been the subject of intense study, particularly in arabidopsis. There are 10 CESA isoforms in arabidopsis (Carroll and Specht 2011) and it has been shown that three different CESAs are required to form a functional CSC in a 1:1:1 ratio (Gonneau *et al.* 2014). Genetic studies have revealed that CSCs in primary cell walls comprise a particular combination of CESAs (Desprez *et al.* 2007; Persson *et al.* 2007) that differs from the CSCs of secondary cell walls (Taylor *et al.* 2003).

The primary cell walls of grasses are also composed of a highly organized network of polysaccharides. However, the non-cellulosic fraction differs significantly between grasses and eudicots in terms of the relative abundance and type of polysaccharides (Carpita 1996; Vogel 2008). In grasses, such as brachypodium, cellulose represents a third of the primary cell wall, but the surrounding matrix glycans largely comprise glucuronoarabinoxylans, with some of the arabinosyl residues being esterified with ferulate, as well as mixed linkage glucans (1,3 1,4-β-glucans) but relatively little xyloglucan or pectin is present (Vogel 2008).

Current understanding of cellulose biosynthesis in grasses is less detailed, but they have conserved CESA clusters, indicating commonalities in the mechanism of cellulose biosynthesis in eudicots and grasses (Handakumbura *et al.* 2015). However, it is notable that a highly potent cellulose biosynthesis inhibitor, isoxaben, has little effect on grasses (Brabham and DeBolt 2013). This compound targets primary cell wall CESA proteins in eudicots (Scheible *et al.* 2001; Desprez *et al.* 2002; Harris *et al.* 2012) and so the resistance observed in grasses suggests that structure-functional differences may exist in the cellulose biosynthetic machinery giving rise to primary cell wall formation.

Prior studies of CESA clade members in arabidopsis revealed that some CESA gene mutations can be tolerated, whereas others are lethal, indicating where redundancy is present (e.g. Persson *et al.* 2007). In addition, point mutations in *AtCESA1* and *-3* have been useful for structure-function predictions (Sethaphong *et al.* 2013). An array of functional genomic tools are now available for brachypodium (Vogel 2010; Brutnell *et al.* 2015), creating opportunities to conduct investigations that were previously restricted to arabidopsis. An example of such an approach is reverse genetics via Targeting Induced Local Lesion IN Genomes (TILLING; McCallum *et al.* 2000; Henikoff *et al.* 2004), which enables the isolation of point mutations in a gene product of interest. Identifying TILLING mutants from within a mutagenized seed population can be accelerated by combining the polymerase chain reaction (PCR) amplification of a gene region of interest with next-generation sequencing (NGS). This approach is referred to as SCAMPRing (sequencing candidate amplicons in multiple parallel reactions; Gilchrist *et al.* 2013).

The CESA1 gene in grasses is proposed to be an ortholog of *radially swollen1* (*rsw1* or *AtCESA1*; Arioli *et al.* 1998; Handakumbura *et al.* 2015), an arabidopsis gene that plays a crucial role in the production of cellulose in the primary cell wall. Here, we sought to gain functional insights into the role of CESA1 in grasses using both TILLING and SCAMPRing. We show that a single missense mutation in this gene results in a significant reduction in the cellulose content of brachypodium and we use molecular dynamic simulation to predict how this mutation alters the dynamics of the CSC. We also discuss the significance of an apparent adaptive anatomical response in the stem of the mutant that occurs as a consequence of the cellulose deficiency.

## Materials and Methods

### Plant material and growth

Seeds were sterilized with 30 % household bleach for 15 min and subsequently washed three times with sterile distilled water and kept at 4 °C for 2 days or 3 weeks. The 3-week cold treatment sufficiently vernalized seeds to promote rapid flowering. For all measurement studies, plants were pre-germinated and seedlings with a protruding radicle < 1 mm were selected for use. To measure coleoptile (dark grown) or root (light grown) length at 7 days after germination, seedlings were placed on agar (11 g L^−1^) plates and grown vertically in growth chambers at 22 °C with a 14-h photoperiod. Plates of dark grown plants were wrapped in aluminium foil. After 7 days, organ length was measured. Seedlings were left in the growth chamber for an additional week and transferred to soil pots and growth was maintained under 24-h supplemental lighting at room temperature. Plants were also grown in subsequent generations under greenhouse conditions (Lexington, KY, USA).

### TILLING by sequencing

The brachypodium line Bd21-3 was used in all experiments. Approximately 10 000 Bd21-3 seeds were mutagenized with exposure of 80 mM ethyl methane sulfonate in a fume hood at room temperature for 16 h. M1 seed were rinsed five times in sterile distilled water and sown in flats of Metromix 360. After an overnight treatment of dark imbibition at 4 °C, flats were placed in a growth chamber at 24 °C/18 °C (day/night) with a 20/4 light/dark cycle. M2 seed from ~5000 M1 plants was harvested for the TILLING population. To identify mutations in the *BdCESA1* gene DNA was collected from 3840 M2 plants in 8 × pools resulting in 480 pools of DNA arrayed in 40 × 96 well PCR plates. Primers were designed to a conserved region of *CESA1—FOR*-AAACGCTTTGGCCAGTCTCCGATATTT and REV-CCACCAGGTTAATCACAAGCACAGTGG **[see****Supporting Information—Table S1****]** using the web-based tool CODDLE (Codons Optimized to Discover Deleterious Lesion; Henikoff *et al.* 2004). PCR reactions were performed on each DNA pool (480 PCR reactions) to maintain low complexity of the pools and ensure amplification of DNA isolated from all individuals that were sampled for each pool. These PCR products were then pooled in row/column arrays (24 + 20) to create 44 superpools. The DNA from the superpools was purified and equal concentrations of DNA fragmented with NEBNext dsDNA Fragmentase (M0348S). The fragments were end-repaired and ligated to adapters for PCR amplification. PCR amplification and multiplexing were performed with the Illumina universal primer and a unique indexing primer to enable deconvolution of pools following sequencing. Equal amounts of DNA from each of the 44 samples were added to a single pool that was sequenced on one lane of a Illumina HiSeq SE.

### Bioinformatic analysis and pool deconvolution

Quality assessments of Illumina reads were performed (FASTQC), adapters trimmed (Trim Galore) and reads mapped to amplicon sequences. Single nucleotide polymorphisms were detected independently in row and column pools to identify intersections that defined unique DNA sample pools comprised of DNA from eight individuals. M3 seed corresponding to each of these M2 progenitors was then grown and DNA isolated from individual plants for Sanger sequencing.

### Identification of brachypodium CESAs

The protein sequences of Arabidopsis and *Oryza sativa* (rice) CESAs were used to search the brachypodium predicted proteome (https://phytozome.jgi.doe.gov/pz/portal.html) using BLAST (Altschul *et al.* 1997) and putative *BdCESA* sequences were searched for CESA-specific glycotransferase domains (Carroll and Specht 2011). Handakumbura *et al.* (2015) named BdCESA genes after their closest Arabidopsis orthologs. We conducted a phylogenetic analysis in Mesquite (100 bootstraps) (Maddison and Maddison 2018) using the class-specific protein region (D to QxxRW motif) from arabidopsis and brachypodium to confirm their results.

### Sectioning

Tissue sections were produced using a vibratome as described by Zelko *et al.* (2012). Sections were stained with were treated with ammonium hydroxide and the fluorescence from ferulate and lignin in the cell walls was observed with an Olympus FV1000 laser scanning confocal microscope using a ×10 N.A. objective.

### Integrated modelling approach

The homology model was refined using all-atom molecular dynamics using AMBER 16 software suite (Case *et al.* 2018) with FF14 protein variant force field and TIP3P water model. Molecular dynamics simulations allow a high resolution of protein conformation states and relate to their energy landscapes. Generally, the atomistic models of large proteins are trapped in local minima, which could limit the scope of equilibrium dynamics. In this study, to enhance sampling, a hyper dynamics approach was implemented, where a harmonic boost is provided to the potential energy function (protein force field), thereby smoothing the potential energy surface (Miao *et al.* 2015). This allows accelerated transitions between low energy states, together with an accurate free energy profile. The simulation protocol included conventional molecular dynamics (cMD) stages of 1000 step minimization using the conjugate gradient and the steepest descent solute constrained isothermal-isobaric simulations. The Gaussian accelerated molecular dynamics (GaMD) module involved 200 000 steps of cMD for equilibration followed by additional 1 × 10^6^ steps of cMD to obtain statistical information about potential energy, which is required for determining boost potential. After adding boost potential, the system was equilibrated for 200 000 steps. Next 1 000 000 steps were used to obtain Gaussian acceleration parameters, the threshold potential and the scaling factor. The upper limit of the standard deviation of the total potential boost and dihedral boost was set to the recommended value of 6.0 kcal mol^−1^. The Particle Mesh Ewald (PME) (Darden *et al.* 1993) summation method was used to calculate the electrostatic potential under periodic boundary conditions applied in all directions. The non-bonded interactions were cut at 9 Å with 0.00001 tolerance of Ewald convergence. The temperature was maintained at 300 K using a Langeven thermostat. The simulations were run for 300 ns for each sample of class specific region (CSR). The protein structure form with the lowest free energy basin was extracted and a DMPC lipid bilayer was constructed around the transmembrane region using CHARMM membrane builder GUI (Jo *et al.* 2008). An all-atom conventional molecular dynamic simulation as performed using AMBER 16 software suite (Case *et al.* 2018) with FF14 protein variant force field and TIP3P water model and Lipid17 force field. The cpptraj module and in-house scripts were used to perform post-processing of the simulation data.

### Expression of putative CESAs

For qRT-PCR, we followed the methods of Udvardi *et al.* (2008). During harvest, shoot tissue (coleoptile removed) was only harvested if the first leaf had not developed a collar, and for elongating coleoptile tissue the encapsulated shoot was removed. mRNA was extracted from each sample using an RNAeasy kit (Qiagen). Amplification cycles and primers are listed in **Supporting Information—Table S1**. Data were transformed to meet basic ANOVA assumptions. Mean values were separated at an alpha value of 0.01 using Tukey’s honest significant difference (HSD) (Abdi and Williams 2010) and back-transformed.

### Instron biomechanical phenotyping

Sections of dried brachypodium stems were taken from lower, middle and upper internodes and analysed using an Instron tabletop load frame (Instron series 3340, clamp model 2710-203: Norwood, MA, USA). The samples were stretched at a rate of 3 mm min^−1^ until they snapped. Resistance to extension was measured with a 100 Newton force transducer (Instron model: 2519-103). Bluhill 2 software (Instron) was used to calculate the tensile stress and strain at maximum load, as well as the elastic modulus. Data were analysed using R (version 3.3.1) and significance set at *P* 0.05 using Tukey’s (Abdi and Williams 2010).

### Cell wall analysis

Senesced plants were harvested and leaf, sheath and stem samples were dried for 1 week at 60 °C. Tissue was either milled or sectioned (to ~3 mm sections) with a scalpel. To obtain alcohol-insoluble residue (AIR), corresponding to crude cell wall, tissue samples we rewashed with 70 % ethanol and placed in a 70 °C water bath for 1 h. This was repeated twice, except that the final ethanol wash was over night, followed by a brief acetone wash at room temperature. Sugars from non-cellulosic polysaccharides in the cell walls were quantified by the method of Foster *et al.* (2010) using AIR (3–5 mg). Neutral sugars (fucose, rhamnose, arabinose, galactose, glucose, mannose, xylose) were identified and quantified by pulsed electrochemical detection using a Dionex ED50 apparatus (Thermo Fisher Scientific). Sugars were separated using a CarboPAC-PA1 anion-exchange column as previously described (Endler *et al.* 2015). Cellulose was quantified colourimetrically using the anthrone-sulfuric acid method (Foster *et al.* 2010). Acid-soluble lignin, acid-insoluble lignin and ash were measured using the laboratory analytical protocols NREL, LAP-004.

## Results

### Identification of *Brachypodium* primary cell wall CESAs

**Figure 1.**
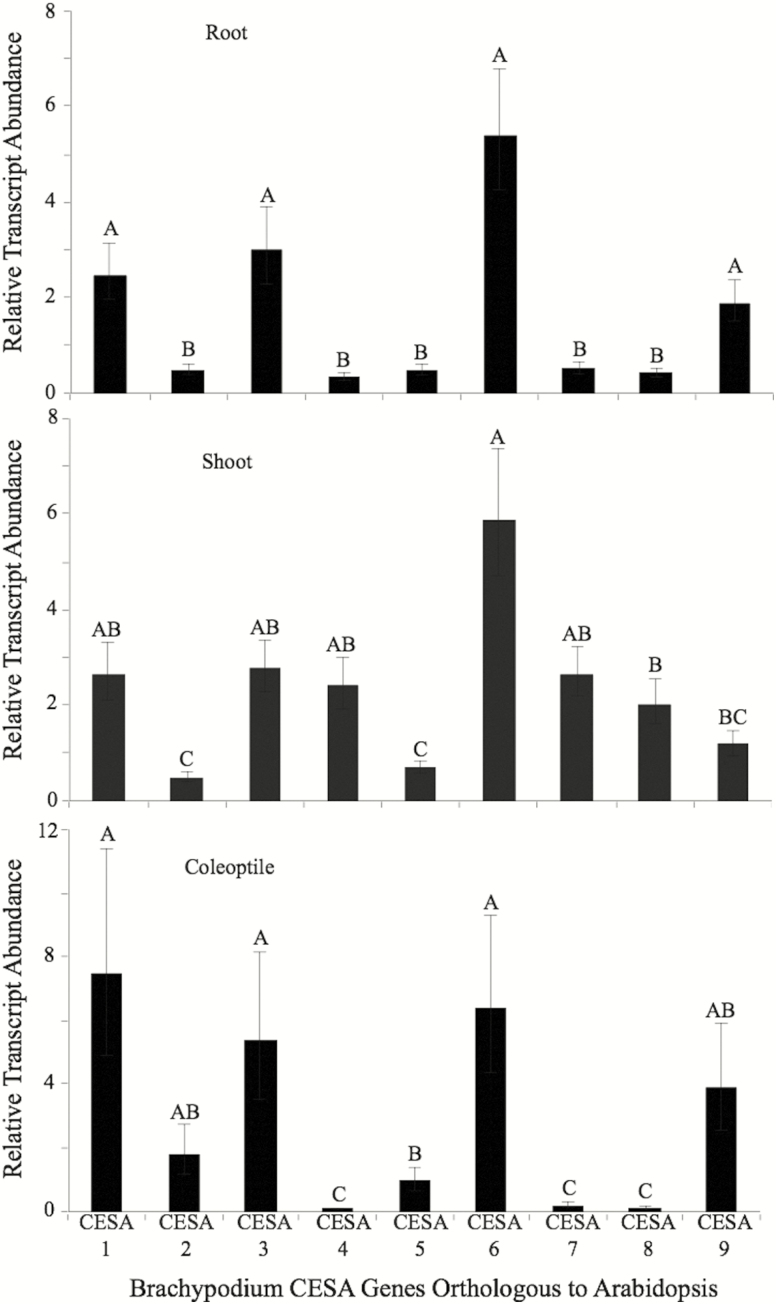
Characterizing relative transcript abundance of *BdCESA* genes in 3- to 4-day-old roots, shoots and coleoptiles to determine primary cell wall CesA. Fold-change values were determined by comparing against gene expression in 3-week-old stem tissue. Means followed by a different letter within a tissue type are considered significantly different at alpha 0.05 using Tukey’s test.

**Figure 2.**
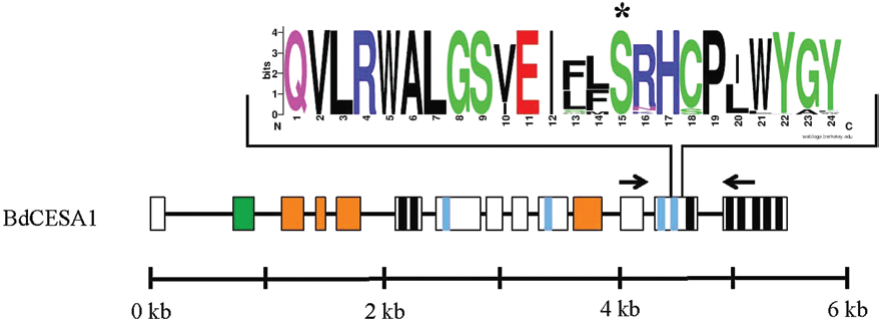
Gene structure, protein topology, TILLING region and conserved sequence logo of point mutation in *BdCESA1*. Sequence logo plot of 88 eukaryotic *CESAs* following the QXXRW motif. The height of a logo is proportional to the conservation frequency at that position. The asterisk is the residue change in the *cesa1*^*S830N*^ mutant sequence. Gene structure and protein topology of plant *CESAs*. The black line and boxes are introns and exons, respectively. Coloured boxes or lines within a box represent unique *CESA* protein domains: zinc finger (green box), class-specific region (orange boxes), black lines (transmembrane domains), catalytic domains D, D, D, QxxRW (blue lines). The black arrows indicate the location of TILLING forward and reverse primers. The scale represents the length of *CESA* gene in kilobase pairs.

A combined phylogenetic and quantitative real-time PCR approach was used to identify *BdCESA* genes involved in primary cell wall cellulose biosynthesis in root, shoot and leaf tissues (Fig. 1). The brachypodium reference genome has 10 predicted *CESA* genes, although *BdCESA10* (Bradi1g36740) is not predicted to have catalytic residues required for glucosyltransferase activity (Morgan *et al.* 2013; Nixon *et al.* 2016; Purushotham *et al.* 2016) and so we did not consider it to be a candidate functional *CESA* gene. It is also worth noting that Bd*CESA5* (Bradi1g29060) does not have a predicted zinc finger domain, believed to be involved in CESA oligomerization (Kurek *et al.* 2002), but it was not excluded from these analyses. The brachypodium *CESA* naming system described by Handakumbura *et al.* (2015) was adopted. They classified *BdCESA* genes based upon their closest arabidopsis orthologs and our data supported their findings. To further validate the phylogenetic predictions, the relative gene expression profiles of *CESA* genes were measured in etiolated coleoptiles (Fig. 1). Since the focus of the current study was primary cell wall *CESA* genes, we evaluated the relative fold change in *CESA* in actively growing tissues to seek *CESAs* that were expressed in all tissues. The relative expression profile of *CESA* genes showed that *BdCESA1* (Bradi2g34240), *BdCESA3* (Bradi1g54250) and *BdCESA6* (Bradi1g53207) were expressed in target tissues (Fig. 2). *BdCESA9* (Bradi1g36740), *BdCESA2* (Bradi1g04597) and *BdCESA5* were detectable but showed insufficient expression uniformity for further evaluation. *BdCESA4* (Bradi3g28350), *BdCESA7* (Bradi4g30540) and *BdCESA8* (Bradi2g49912) were the subject of an extensive prior study and are known to be required for cellulose biosynthesis in secondary cell walls (Handakumbura *et al.* 2015). As expected, their expression was significantly reduced in coleoptile and root tissue (Fig. 1).

Based on these findings, and in accordance with Handakumbura *et al.* (2015), *BdCESA1, 3,* and *6* expression was consistent with their having a role in primary cell wall cellulose biosynthesis. Genetic evidence from arabidopsis suggests that AtCESA1 and 3 are required for cellulose biosynthesis (Arioli *et al.* 1998; Kurek *et al.* 2002; Persson *et al.* 2007) and we sought to understand the functional genetic significance of primary cell wall *CESAs* in grasses via TILLING for a mutation in *BdCESA1*.

### Targeting and identification of *BdCESA1* TILLING mutant

Screens were conducted on an ethyl methane sulfonate (EMS)-mutagenized population of brachypodium accession Bd21 (see Materials and Methods). In brief, primers were designed to genomic regions with the highest probability for EMS-induced missense and nonsense lesions in Bd*CESA1*, using the web-based tool CODDLE (Codons Optimized to Discover Deleterious Lesion; McCallum *et al.* 2000; Henikoff *et al.* 2004; Gilchrist *et al.* 2013). A 1096-bp region of the *BdCESA1* gene was selected for TILLING (see Materials and Methods), corresponding to approximately one-sixth of the full-length genomic sequence. This DNA region encodes the last half of the glycosyltransferase domain to the sixth transmembrane domain (Fig. 2, black arrows indicate primer location). To identify point mutations, primers were used to amplify the region of interest, using pooled DNA samples from our TILLING population as a template. Next-generation sequencing of these samples revealed a point mutation, which was predicted to result in an asparagine instead of a serine at position 830 of the BdCESA1 protein. No further predicted or sequenced mutations were identified in *BdCESA1*.

The *Bdcesa1*^*S830N*^ is located in the cytosolic catalytic loop 10 amino acids downstream of the QXXRW motif and ~23 amino acids before the beginning of the third transmembrane alpha helix (Fig. 2, asterisk). A sequence analysis of plant CESA1 proteins based on the 19 amino acids following the QXXRW motif revealed high conservation within this region (Table 1). A total of 88 plant CESA proteins were used to generate the sequence logo plot (Fig. 2), where the height of a logo is proportional to the frequency at which it occurs at that position (Schneider and Stephens, 1990). Analysis of the sequence logo plot revealed the *Bdcesa1*^*S830N*^ would change the conserved serine found in all identified CESAs to an asparagine.

**Table 1.** Sequence alignment of various isoforms showing conserved region E(K/R)xFGxS.

BdCESA1 --------RMMKRTESSAPIFNMEDIEEGIE--GYEDERSMLMSQKRLEKRFGQSPIFTA BdCESA3 --------KSNKHVDSSVPVFNLEDIEEGVEGAGFDDEKSLLMSQMSLEKRFGQSAAFVA BdCESA6 --------LFFKRAENQSPAYALGEIEEGIPGA--ENDKAGIVNQEKLEKKFGQSSVFAA BdCESA9 ----------LRRTMSVVPLLESEEDEEGIAEGGR--RRRLRSYSAALERHFGQSPLFIA BdCESA4 KDKLGGAPKKGGSYRKQQRGFELEEIEEGIEGYD-ELERSSLMSQKNFEKRFGQSPVFIA BdCESA7 -----GLP---------------ESVGDGMDG-----DKEMLMSQMNFEKRFGQSAAFVT BdCESA8 --------RDSRREDLESAIFNLREIDNY-----DEYERSMLISQMSFEKSFGQSSVFIE . : : . : * : * * * * *

### Expression of *CESA1* in Bd*cesa1*^*S830N*^

An important question related to the gene of interest was whether the S830N missense substitution influenced expression of the *BdCESA1 in planta*. The region of interest is near the catalytic domain, but the missense mutation was not predicted to result in a premature stop codon or alter *BdCESA1* gene expression. The expression of the native *BdCESA1* was evaluated in three biological replicates of the *Bdcesa1*^*S830N*^ compared with wild-type. Using semi-quantitative PCR (25 cycles using GAPDH as a control gene) we found no change in gene expression in expanding shoot tissue **[see****Supporting Information—Fig. S1****]**. These data support the prediction that the missense mutation will not interfere with *BdCESA1* gene expression, but might lead to structural differences in the translated gene product.

### Relative changes in cellulose content in *cesa1*^*S830N*^

**Figure 3.**
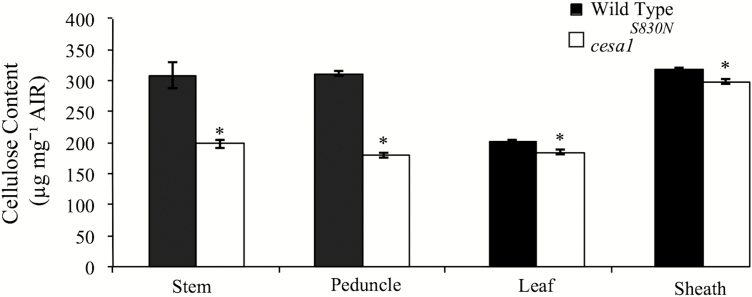
Cellulose content of stem, peduncle, leaf and sheath, from mature and senesced wild-type (black) and *Bdcesa1*^*S830N*^ mutants. A two-tailed *t*-test was used to compare means (±1 SE) within a tissue type and an asterisk indicates a significantly difference at an alpha value of 0.05.

Cellulose content was measured in leaf, sheath, stem and peduncle tissue of mature wild-type (Bd21-3) and *Bdcesa1*^*S830N*^ plants, and a significant reduction was found in all mutant samples. On average, *Bdcesa1*^*S830N*^ mutants had 7 % less cellulose in leaf and sheath tissue and 25 % less cellulose in stem and peduncle tissues compared to wild-type (Fig. 3, significance established via Tukey’s mean separation *P* > 0.05).

### Mutant phenotypes associated with reduced cellulose content

**Figure 4.**
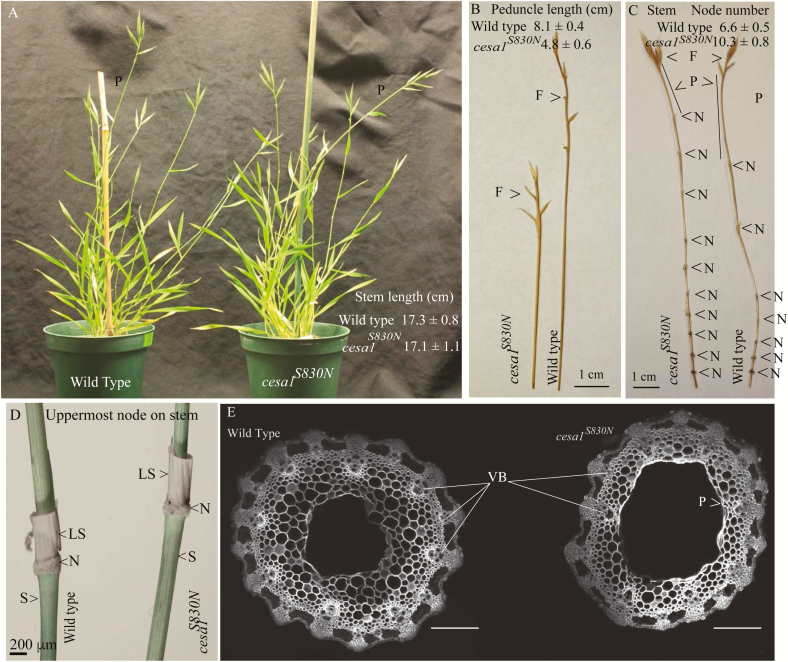
Morphological characteristics of wild-type and the Bd*cesa1*^*S830N*^. (A) Wild-type and the *Bdcesa1*^*S830N*^ plants were propagated side by side and plant height and the image was captured during seed fill growth stages (P, peduncle). (B) Representative sample of stem tissue (right) and peduncle (left) demonstrates nodes frequency and peduncle length differences (N node; P peduncle; and F floret). (C) The morphology of the uppermost node on the stem reveals little change between wild-type and Bd*cesa1*^*S830N*^ (N node; LS leaf sheath; and S stem), note that the leaf blade was excised from the LS. Measurement of coleoptile (dark grown) and root (light grown) length after 7 days and peduncle length for each genotype. A two-tailed *t*-test was used to compare means (±1 SE) of growth characteristics within a tissue type and an asterisk indicates a significantly difference at an alpha value of 0.05. Scale bars (A) 2.54 cm, (B, C) 1 cm, (D) 200 μm, E 40 μm.

By TILLING in a mutagenized background we identified a single allele *Bdcesa1*^*S830N*^. As noted by Liu *et al.* (2017), *CESA* mutations in grasses are lacking, which complicates comparisons to other alleles. Gene complementation is also made difficult by the fact that the mutant allele likely encodes a functional protein product that can interact with the wild-type product. Therefore, where appropriate, we compared phenotypes of *Bdcesa1*^*S830N*^ with those of the *dwf1-1* from *Sorghum bicolor* (Petti *et al.* 2015). The *dwf1-1* linked to an insertion mutation in *gibberellin20 (GA20)-oxidase* and *Bdcesa1*^*S830N*^ alleles displayed reduced stem cellulose content (35 and 28 %, respectively) and homozygous lethality. In eudicots, the loss of this vital component of the cell wall leads to stunted plants, in which root and shoot tissue expansion is severely reduced (Arioli *et al.* 1998). We observed a similar phenotype in the peduncles of *Bdcesa1*^*S830N*^ mutants, with a 41 % reduction in length compared to wild-type peduncles (*P* < 0.001, Student’s *t*-test; Fig. 4A and B). However, the stem lengths of *Bdcesa1*^*S830N*^ mutants were similar to their wild-type counterparts (Fig. 4A), as were coleoptiles (wild-type 3.7 cm ± 0.2; *Bdcesa1*^*S830N*^ 3.6 cm ± 0.2), which is atypical of the normal dwarfism seen in arabidopsis *rsw1-1* (Arioli *et al.* 1998). We therefore examined other stem characteristics that could be associated with lower cellulose content.

To determine whether the mutation affected the biomechanical properties of the stems, we measured the elastic modulus of isolated and dried lower, middle and upper stem sections, using an Instron materials testing instrument. These three regions were chosen for comparison because lower stems have secondary cell walls, whereas upper stems have predominately primary cell walls. There was no significant difference in the elastic modulus values for the lower and middle sections (*n* = 4, *P* > 0.05, Tukey’s HSD). However, we detected a highly significant reduction in the elastic modulus in the upper stem of *Bdcesa1*^*S830N*^ (1576 ± 386 SD, *n* = 9) compared with controls (716 ± 269 SD, *n* = 15, *P* = 0.009). Note that with Young’s modulus, the larger number equates to greater force needed to stretch a substrate (less elastic). In addition, the tensile strength of the lower and middle sections of the Bd*cesa1*^*S830N*^ stems was not significantly different to control (*n* = 3, *P* > 0.05) but the upper stems (60.6 ± 10.6 SD) were significantly weaker than the control at (86.9 ± 28.7 SD, *P* = 0.003). Given that cellulose microfibrils act as the major load-bearing component of the wall, these data indicate a functional association between the *Bdcesa1*^*S830N*^ allele and loss of structural integrity in the primary cell wall.

The stems of *Bdcesa1*^*S830N*^ were found to be both deficient in cellulose, as well as structurally weaker and less stiff, and yet the mutants grew to the same height as wild-type plants. We therefore examined plant morphology and found that the stems of *Bdcesa1*^*S830N*^ mutants have a significantly higher mean density of nodes (10.3 ± 0.8) (Fig. 4C) compared with wild-type (6.6 ± 0.5) (two-tailed *t*-test, *P* > 0.001, *n* = 7). Correspondingly, significantly reduced internode lengths were observed but these differences were more pronounced in the upper stem internode distances. For instance, in wild-type, the lower three internodes had a mean length of 9.3 cm ± 2.5, whereas *Bdcesa1*^*S830N*^ displayed average internode length of 7.0 cm ± 1.2. In contrast, the upper three internode lengths were significantly different (wild-type 37.3 cm ± 0.96 compared to *Bdcesa1*^*S830N*^ 21.7 cm ± 2.2, *n* = 4). The reduced internode and peduncle length phenotypes were observed in plants growing in either growth chamber or greenhouse conditions (Fig. 4C). To corroborate these data, we examined node frequency in the Sorghum *dwf1-1* allele (Petti *et al.* 2015). Here, node number was again significantly greater in *dwf1-1* compared to wild-type control plants (mean of 10.3 ± 0.6 for *dwf1-1* versus 7.8 for control). This was accompanied by severe reduction in internode expansion. It should be noted that *dwf1-1* is homozygous lethal and displays a greater reduction in cellulose than *Bdcesa1*^*S830N*^; thus, results could reflect the severity of this mutation. We carefully examined the morphology of the uppermost node and observed no gross anatomical difference between wild-type and *Bdcesa1*^*S830N*^ (Fig. 4D). Transverse sections were prepared from mature peduncles, which showed the greatest length reduction. No collapsed vascular xylem was observed (Fig. 4E) in wild-type or Bd*cesa1*^*S830N*^ but modest aberration in the integrity of the parenchyma layer and thickness of the cortex were distinguishable.

### Non-cellulosic sugars in *Bdcesa1*^*S830N*^

Of the sugars released from the non-cellulosic polysaccharides in the cell walls, negligible changes (1.2-fold increase or decrease) were observed in *Bdcesa1*^*S830N*^ compared to wild-type (Table 2). The exception was galactose, which exhibited an ~30 % greater relative abundance in stems and sheaths of the mutant plants. While a trend towards modest increases in the lignin content was observed in the *Bdcesa1*^*S830N*^ mutant stems (2.0 ± 0.2 % soluble and 20.4 ± 0.9 % insoluble), these differences were not significant compared with wild-type (1.91 ± 0.2 % soluble and 19.5 ± 0.9 % insoluble) (*n* = 4, *P* > 0.05, two-tailed *t*-test).

**Table 2.** Quantification of non-cellulosic trifluoracetic acid-soluble sugars in the stem, sheath and leaf of wild-type and TILLING mutants. ^a^Tissue from six biological reps was measured in triplicate for each genotype for neutral sugars. ^b^Fucose and mannose values were either less than 0.7 % or not detectable in tissue and not shown. ^c^A two-tailed *t*-test was used to compare means (±1 SE) of sugars within a tissue type and an asterisk indicates a significantly difference at an alpha value of 0.05. All values are rounded to the nearest 1 or 10th.

	Rhamnose^b^	Arabinose	Galactose	Glucose	Xylose
Stem^a^					
Wild-type	0.8 ± 0.1^c^	16 ± 0.4	3.2 ± 0.1	20 ± 0.7	60 ± 0.9
*cesa1*^*S830N*^	0.9 ± 0.1*	16 ± 0.5	4.3 ± 0.2*	21± 0.6	58 ± 0.7*
Sheath					
Wild-type	1.1 ± 0.1	20 ± 1.0	5.0 ± 0.2	18 ± 0.4	56 ± 1.0
*cesa1*^*S830N*^	1.2 ± 0.1	20 ± 0.6	6.4 ± 0.3*	17 ± 0.6	55 ± 1.3
Leaf					
Wild-type	2.3 ± 0.1	19 ± 0.6	7.6 ± 0.4	27 ± 1.7	44 ± 2.0
*cesa1*^*S830N*^	1.8 ± 0.1	21 ± 0.5*	7.5 ± 0.2	23 ± 0.8*	47 ± 1.0

### Biochemical modelling of the *Bdcesa1*^*S830N*^ into a 3D atomistic model of cellulose synthase

**Figure 5.**
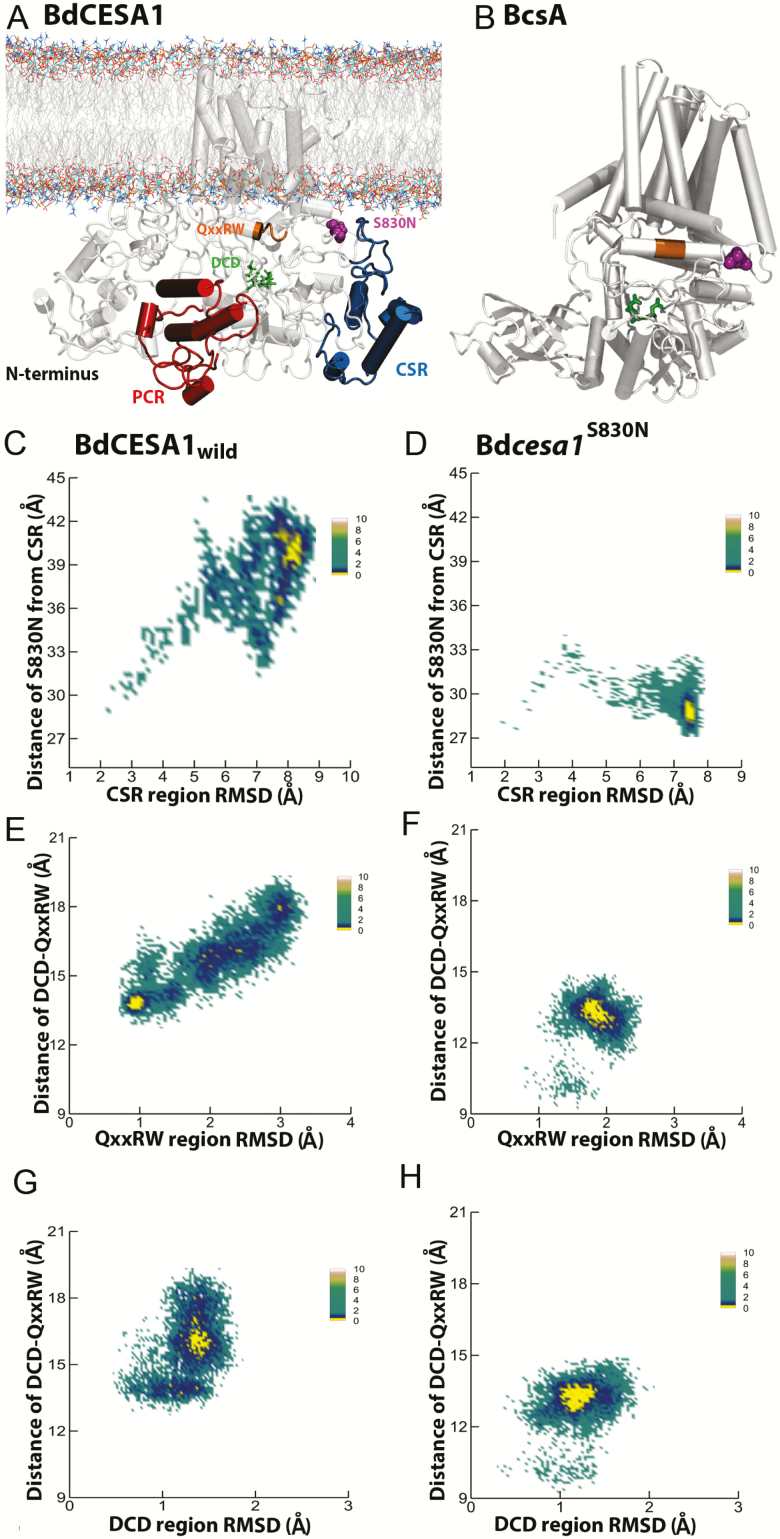
The location of S830 in the BdCESA1 model (A) and the BscA model (B). The mutation site is coloured magenta, DCD motif is green and QxxRW is orange. In BdCESA1 model the PCR is red and CSR is blue. BscA does not have PCR and CSR. (C, D) Conformational sampling of the CSR region as function of distance from mutation site and root-mean-square deviation for wild-type and mutant, respectively. (E, F) Conformational sampling of QxxRW region as a function of distance from the DCD site and root-mean-square deviation for wild-type and mutant, respectively. (G, H) Conformational sampling of the DCD region as function of distance from the QxxRW and root-mean-square deviation for wild-type and mutant, respectively. Conformational spaces (C–H) are coloured based on free energy with yellow being lowest energy state.

The experimental determination of plant CESA structures presents a technical challenge due to the large size of the proteins, the fact that they contain eight membrane spanning domains and contain a relatively high proportion (~15 %) of intrinsically disordered regions (Scavuzzo-Duggan *et al.* 2018). Part of the PCR region of OsCesA8 from rice (*O. sativa*) has been solved using X-ray crystallography (Rushton *et al.* 2017) and the configuration of AtCesA1 CatD region was determined using small angle X-ray scattering (SAXS) (Vandavasi *et al.* 2016). Additionally, the solution structure of bacterial cellulose synthase displays some biochemical dissimilarities to plant CESA(s) (Morgan *et al.* 2013). Therefore, standard homology modelling software, such as Phyre2 (Kelley *et al.* 2015), fails to produce robust models of plant CESA proteins **[see****Supporting Information—**Fig. S2**]**. Here, we used the GhCESA model template and SWISS-MODEL (Biasini *et al.* 2014) to build a 3D model of BdCESA1 to investigate possible structural changes occurring due to the amino acid substitution at S830. The complete model of BdCESA1 is shown in Fig. 5A.

Results suggest that Bd*cesa1*^*S830N*^ is on the solvent-accessible side of CESA and that S830 is predicted to interact with the CSR. When incorporating the mutation into the model we see that the migration of the CSR domain to the membrane is inhibited (Fig. 5). The 3D structure highlighted components of the catalytic core in orientations of PCR, CSR, QxxRW and DCD relative to mutation site S830N (Fig. 5). Simulation results predict that the mutation might cause structural alterations in the CSR region. Further, maps of the free energy landscape reveal the conformational space defined by the root-mean-square deviation of the CSR region and the distance of the CSR from the S830N mutation site (Fig. 5B and C). The energy landscapes include local metastable states separated by small energy barriers and had few ‘lowest free energy’ regions, seen in yellow. Computational results show that in contrast to wild-type CSR, the mutant CSR moves closer to the mutation site and QxxRW region. The CSR is largely unstructured and the prediction of the motion for migration indicates that it is possible **[see****Supporting Information—Fig. S3****]**. Using the functional amino acid Bd*cesa1*^*S830N*^ substitution as a target, we computationally evaluated a range of alternative substitutions (Table 3) at this site. It was computationally predicted that altering S830 to any alternative amino acid would have a deleterious influence on the CSR stability.

**Table 3.** Effect of point mutation predictions with other amino acids.

Variant	PROVEAN score	PROVEAN prediction (cut-off = −2.5)	Duet ΔΔG	DUET prediction
S830A	−2.853	Deleterious	−0.636	Mildly deleterious
S830V	−5.704	Deleterious	−0.074	Mildly deleterious
S830L	−5.701	Deleterious	0.35	Stabilizing
S830G	−3.808	Deleterious	−1.025	Mildly deleterious
S830W	−6.659	Deleterious	−0.924	Mildly deleterious
S830T	−2.853	Deleterious	−0.445	Mildly deleterious
S830Q	−3.805	Deleterious	−0.428	Mildly deleterious
S830E	−3.807	Deleterious	−0.339	Mildly deleterious
S830C	−4.755	Deleterious	−0.245	Mildly deleterious
S830R	−4.756	Deleterious	−0.336	Mildly deleterious
S830P	−4.758	Deleterious	−0.764	Mildly deleterious
S830D	−3.808	Deleterious	−0.54	Mildly deleterious
S830F	−5.705	Deleterious	−0.689	Mildly deleterious
S830I	−5.703	Deleterious	0.327	Stabilizing
S830H	−4.758	Deleterious	−1.604	Mildly deleterious
**S830N**	−**2.855**	**Deleterious**	−**0.619**	**Mildly deleterious**
S830M	−4.752	Deleterious	0.248	Stabilizing
S830Y	−5.706	Deleterious	−0.446	Mildly deleterious
S830K	−3.805	Deleterious	−0.211	Mildly deleterious

**Figure 6.**
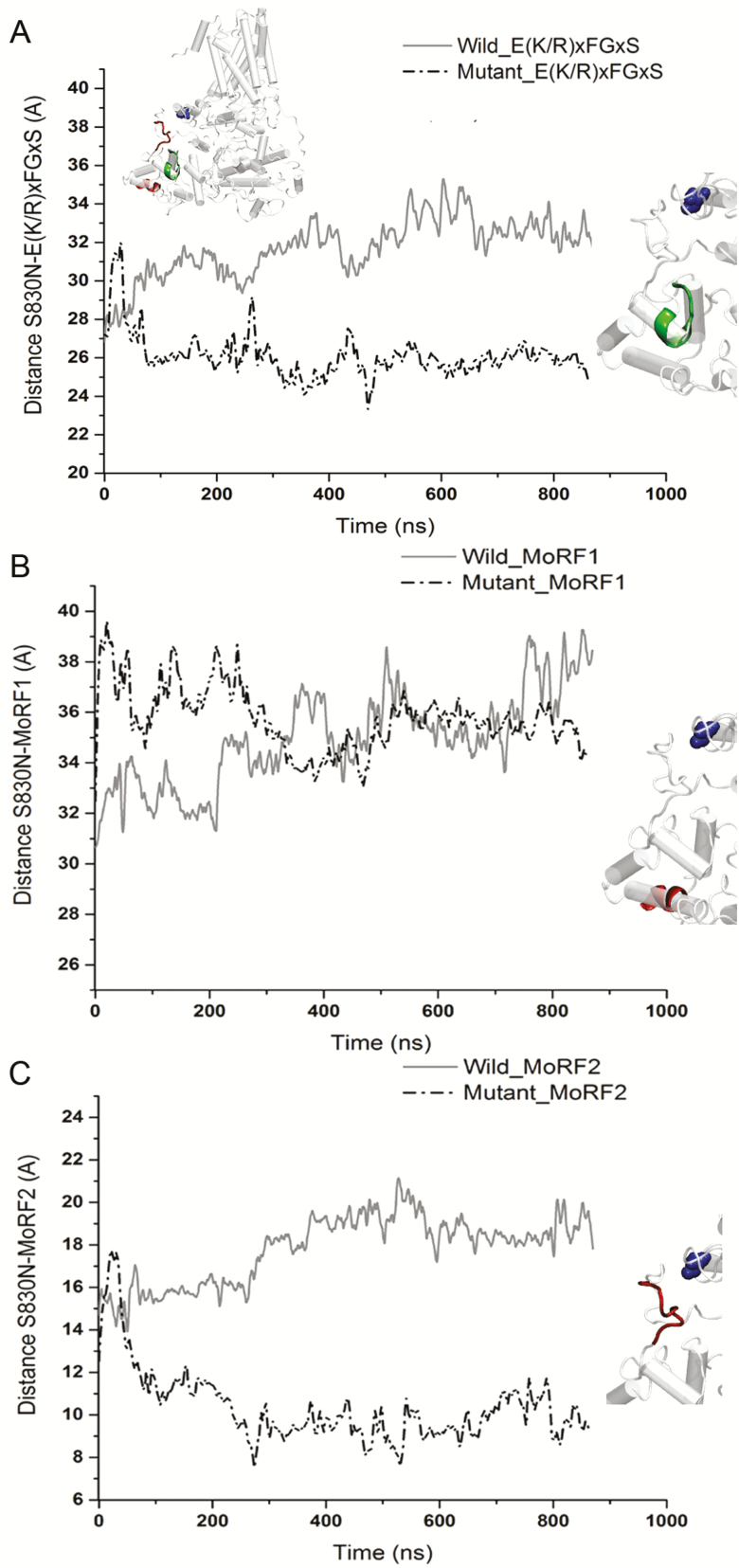
Time evolution of the interactions measured as a proximity distance between S830N site and the elements of the CSR region (A) conserved motif E(K/R)xFGxS. (B) MoRF1, (C) MoRF2 located in the wild-type and the mutant CESA.

We looked further into the structural dynamics of the components of the CSR that may have biological significance related to the S830N point mutation (Fig. 6). In the ancient bryophyte *Physcomitrella patens*, quantification of the hyper-variable nature of CSR identified biologically significant molecular recognition of features (Morfs) that can undergo order–disorder transition (Scavuzzo-Duggan *et al.* 2018). In Fig. 5C and D the entire CSR domain in *Bdcesa1*^*S830N*^ is predicted to shift closer to the mutation site and QxxRW region. More specifically, the conserved and MoRF regions of mutant CeSA CSR were up to 10 Å closer to the mutation site (Fig. 6), while the proximity of MoRF2 to the mutation site in mutant CESA was comparable to that of wild-type. **Supporting Information—Fig. S4** compares the solvent accessible surface area of components of CSR.

### Predicted effect of mutation on the activity of BdCESA1

In Fig. 5, we defined the free energy landscape based on the distance between DCD and QxxRW motifs and the root-mean-square deviations of QxxRW. The mutant CESA (Fig. 5F) exhibited root-mean-square deviations with a tighter distribution. The S830N mutation was predicted to make the QxxRW motif more rigid than its counterpart in the wild-type. In contrast, the mutant DCD motif displayed higher structural deviations (Fig. 5G and H). The lowest energy basins show that DCD and QxxRW motifs were more distant from each other in the mutant CESA. We also compared the deleterious nature of the substitution of serine with other amino acids and found that S830N mutation would be mildly deleterious based on DUET prediction score (Table 3).

## Discussion

Despite the majority of terrestrial biomass being produced by grasses, molecular genetic studies into cellulose biosynthesis are limited. It is worth noting that based on phylogenetic studies (Handakumbura *et al.* 2015), cellulose biosynthetic genes appear quite conserved across grasses and eudicots. There are, however, some curious differences. For instance, it is not known why class-L herbicides, known as cellulose biosynthesis inhibitors, are far more potent against broadleaf plants than grasses. One hypothesis is that differences in cell wall composition between grasses and eudicots allows for modest expansion during cellulose biosynthesis inhibitor stress (Brabham *et al.* 2018). In this study, we used TILLING and SCAMPRing targeting a region adjacent to the catalytic domain in *BdCESA1* because it is predicted to be orthologous to the severe *rsw1* (Arioli *et al.* 1998; Persson *et al.* 2007). Unlike *rsw1*, which was severely dwarfed and null lethal, *Bdcesa1*^*S830N*^ internode expansion was greatly reduced, but normal plant height was reached (Fig. 4A). Brachypodium plants carrying the *Bdcesa1*^*S830N*^ allele displayed an unexpected physiological change that correlated with the lack of expansion and reduced cellulose, which was to increase the number of nodes along the stem (Fig. 4C). Hence, it is feasible that, similar to chemical disruptions of cellulose biosynthesis in grasses with certain class-L herbicides, the mutant with the genetic dysfunction can still grow due to having more nodes (Fig. 4).

Interpretation of complex phenotypes, however, must be tempered by the single allele derived from the EMS population used for TILLING experiments. Results could be linked to secondary mutations giving rise to the secondary phenotypes observed. In support of a possible correlation, the results observed were largely expansion-driven, which is a consistent feature of cellulose deficit in expanding plant tissues (Desprez *et al.* 2007; Handakumbura *et al.* 2015). There are very few examples to cross-reference our observations to. We did re-visit the phenotype of the sorghum *dwf1-1* mutation (Petti *et al.* 2015), which caused severe cellulose deficit linked to a mutation in GA20-oxidase. Here, node density increased along the stem and internode length was shorter, but the *dwf1-1* plant failed to reach the height of the wild-type. This may reflect that *dwf1-1* contained a greater reduction in cellulose than Bd*cesa1*^*S830N*^, which in turn influenced plant expansion akin to a dose effect. Alternatively, it could also be suggested that the *dwf1-1* mutation in GA20-oxidase, imparted a broader influence on expansion and development than targeting cellulose alone. Irrespective of mechanism, it was common to both grass taxa (Panicoideae and Poaceae) that cellulose deficit was accompanied by a increased node number along the stem axis and decreased internode expansion.

Consistent with a phenotype linked to the primary cell wall, the upper stems of Bd*cesa1*^*S830N*^ were biomechanically weaker, but the lower stem tissue exhibited no significant change in biomechanical properties. These data support the suggestion that the *Bdcesa1*^*S830N*^ phenotypes were primary cell wall linked, and that secondary cell wall thickening, which is more developed in the lower grass stem, helped compensate for any biomechanical deficiencies.

The limited number of homologous structures, the presence of intrinsically disordered regions and the low number of solved transmembrane protein structures mean that an integrated modelling approach, using bioinformatics tools and molecular dynamic simulation, can only generate hypothetical structural predictions, although this approach has been used to develop hypotheses and predictions (Sethaphong *et al.* 2013; Slabaugh *et al.* 2014; Lei *et al.* 2015; Nixon *et al.* 2016). While requiring further work and future biochemical validation, we hypothesize that structural changes in the active binding site affected CESA activity. When coupled with the reduced accessibility of MoRFs binding regions in the CSR domain these structural changes would have implications for the assembly of the CSC. Future work is needed to understand the disordered protein region in CESA (Shoemaker *et al.* 2000; Scavuzzo-Duggan *et al.* 2018) as these regions have played diverse biochemical roles (Dunker *et al.* 2005; Mohan *et al.* 2006).

In conclusion, these data provide fundamental information about the nature of cellulose biosynthesis in grasses. Some practical applications may be foreseeable. For instance, stem lodging (breaking of the stem) of major cereal crops is tightly linked to nodal structure and alone accounts for 5–20 % of annual losses in global grain production (Robertson *et al.* 2017). Our data support the notion that primary cell wall cellulose biosynthesis is one of many factors influencing the complex trait of stem morphology and biomechanics (Von Forell *et al.* 2015) and raises the possibility of strength gradients across the grass stem.

## Supporting Information

The following additional information is available in the online version of this article—

**Table S1.** Primers used in all experimental procedures.

**Figure S1.** Semi-quantitative polymerase chain reaction (PCR) assessment of the *BdCESA1* transcript in the wild-type and mutant backgrounds.

**Figure S2.** A computationally derived homology model utilized Phyre2.0.

**Figure S3.** Disorder prediction form ANCHOR probability scores.

**Figure S4.** Comparing solvent accessible surface area of conserved region, MoRF1 and MoRF2 in wild-type and mutant CESA.

plz041_suppl_Supplementary_Information

## Sources of Funding

This research was supported by the United States National Science Foundation (NSF) 1826715 (SD), USDA Hatch Funding (SD) and Department of Energy DOE-FOA 10-0000368 (SD, JKCR, TB). Computational work was supported by The Center for Lignocellulose Structure and Formation, an Energy Frontier Research Center funded by the U.S. Department of Energy, Office of Science, Office of Basic Energy Sciences under Award Number DE-SC0001090.

## Contributions by the Authors

SD, JKCR, YGY and TB conceived and designed the study. CB, AS, JS, YR, IK, and KK collected the data and conducted the statistical analyses. All authors contributed to writing and revising the manuscript.
