# Supplementary material for: Biochemical and physiological flexibility accompanies reduced cellulose biosynthesis in Brachypodium cesa1S830N
Source: AoB Plants. 2019 Jul 13;11(5):plz041. doi: 10.1093/aobpla/plz041 (PMC6795283; doi:10.1093/aobpla/plz041)
Supplement: plz041_suppl_Supplementary_Information [file plz041_suppl_supplementary_information.docx]

**Supplementary online data**

**Biochemical and physiological flexibility accompanies reduced cellulose biosynthesis in brachypodium *cesa1^S830N^***

**Supplemental Table S1.** Primers used in all experimental procedures

| Supplement Table 2. List of primers used in experiment | | | | |
| --- | --- | --- | --- | --- |
| Primer Name | Gene Number | Use | Forward | Reverse |
| CESA1 | Bradi2g34240 | qRT-PCR | TAAGCAAGGCAATGGCAAAGGTCC | ATGTGGTTCATGGCGAGAGGATGA |
| CESA2 | Bradi1g04597 | qRT-PCR | TGACGGCAATGAGCTTCCTCGT | ATGGCGCCAGCTTTCTTGTGGT |
| CESA3 | Bradi1g54250 | qRT-PCR | GGTATCTCCTACGCCATCAACAGTGG | CTGCTTACCCATAAGACCCTTGAGGA |
| CESA4 | Bradi3Gg8350 | qRT-PCR | ACAGGATCGACAAGTGGAAGACGAA | GTCGTTCTTGTCATCGTCATCATCGC |
| CESA5 | Bradi1g29060 | qRT-PCR | GAGAATCCACCCACTTCCTTATG | GGTGCAAACTCTCCTGTTTCT |
| CESA6 | Bradi1g53207 | qRT-PCR | AGCAAGGCAGGGATTGATCTACCA | TTGTCGACTGTATGGGACGTGGAA |
| CESA7 | Bradi4g30540 | qRT-PCR | TGCAAAGTGGGACGAGAAGAAGGA | TCGCCTCGTCGTTTATTGGGACAT |
| CESA8 | Bradi2g49912 | qRT-PCR | TTCGGTTTCCTCTCAGGCCTTTCT | AGTGCCAGCTCATAATTCCAGCGA |
| CESA9 | Bradi1g02510 | qRT-PCR | ACCGTGACAACCAAGGCTGGA | AAATGCCAGCCACTACCCCGA |
| CESA1 | Bradi2g34240 | Tilling | AAACGCTTTGGCCAGTCTCCGATATTT | CCACCAGGTTAATCACAAGCACAGTGG |
| CESA3 | Bradi1g54250 | Tilling | AGAGATTTGGACAGTCCGCAGCTTTTG | TTCCTAGCAGTTGATGCCACAGGTTTG |
| GAPDH | Bradi3g14040 | qRT-PCR | AATAAATGTGGACATTGAGTCGCC | GAAATAGCGGGAGCAACATAGATTAC |
| GAPH intron spanning | cDNA quality check | | ATGGGCAAGATTAAGATCGGAATCAACGG | AGTGGTGCAGCTAGCATTTGAGACAAT |

**Supplemental Figures**

**Supplemental Fig S1**. Semi quantitative PCR assessment of the *BdCESA1* transcript in the wild-type and mutant backgrounds were unchanged as compared with *GAPDH* control gene expression.


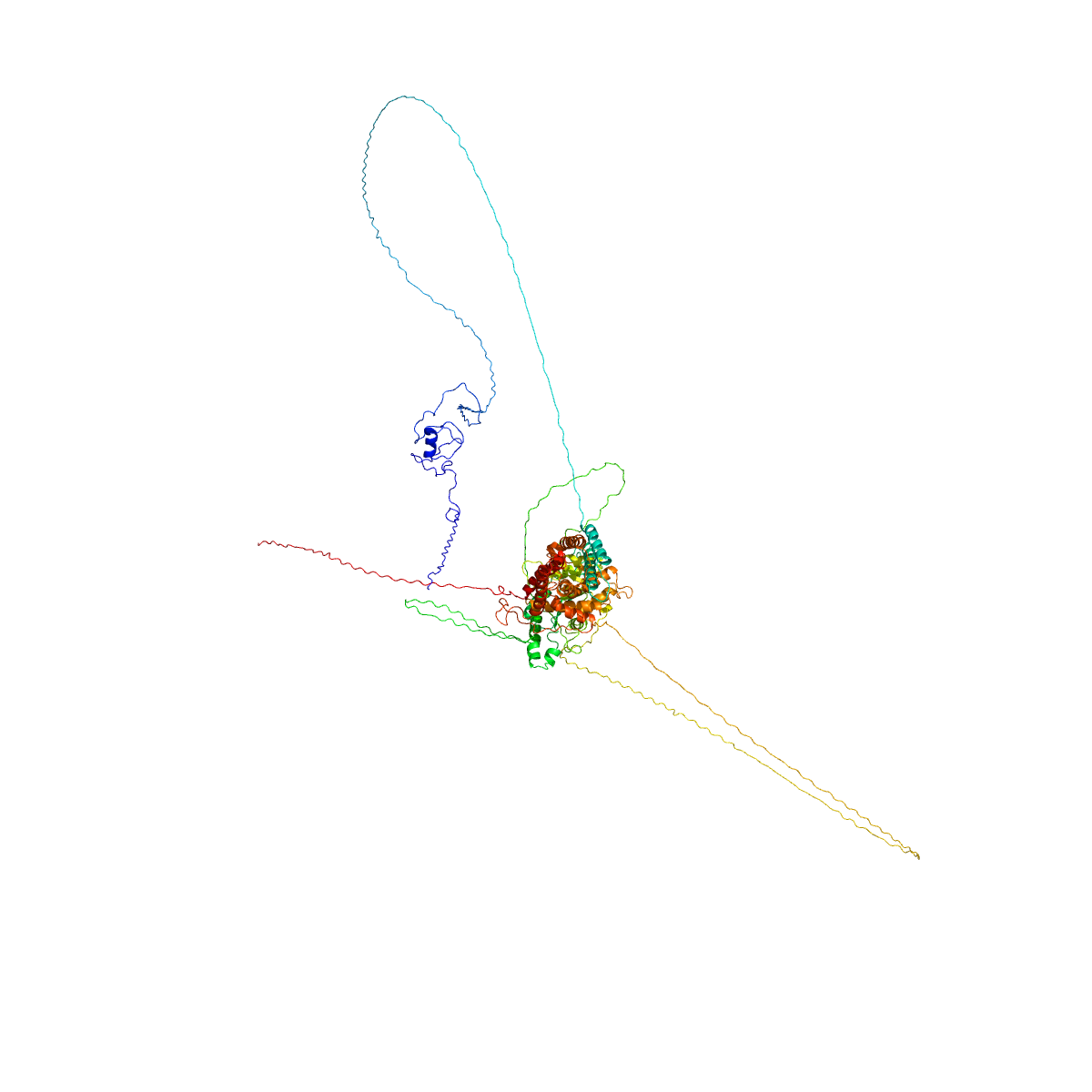


**Supplemental Fig S2** A computationally derived homology model utilized Phyre2.0. The figure shows a resultant homology model of BdCESA derived from the protein databases of known structures. Presence of large unfolded loops (string lines of yellow, light-dark blue, red) indicates poor predictions since there is no experimentally resolved plant synthase protein till date and poor sequence similarity make predictions even challenging.


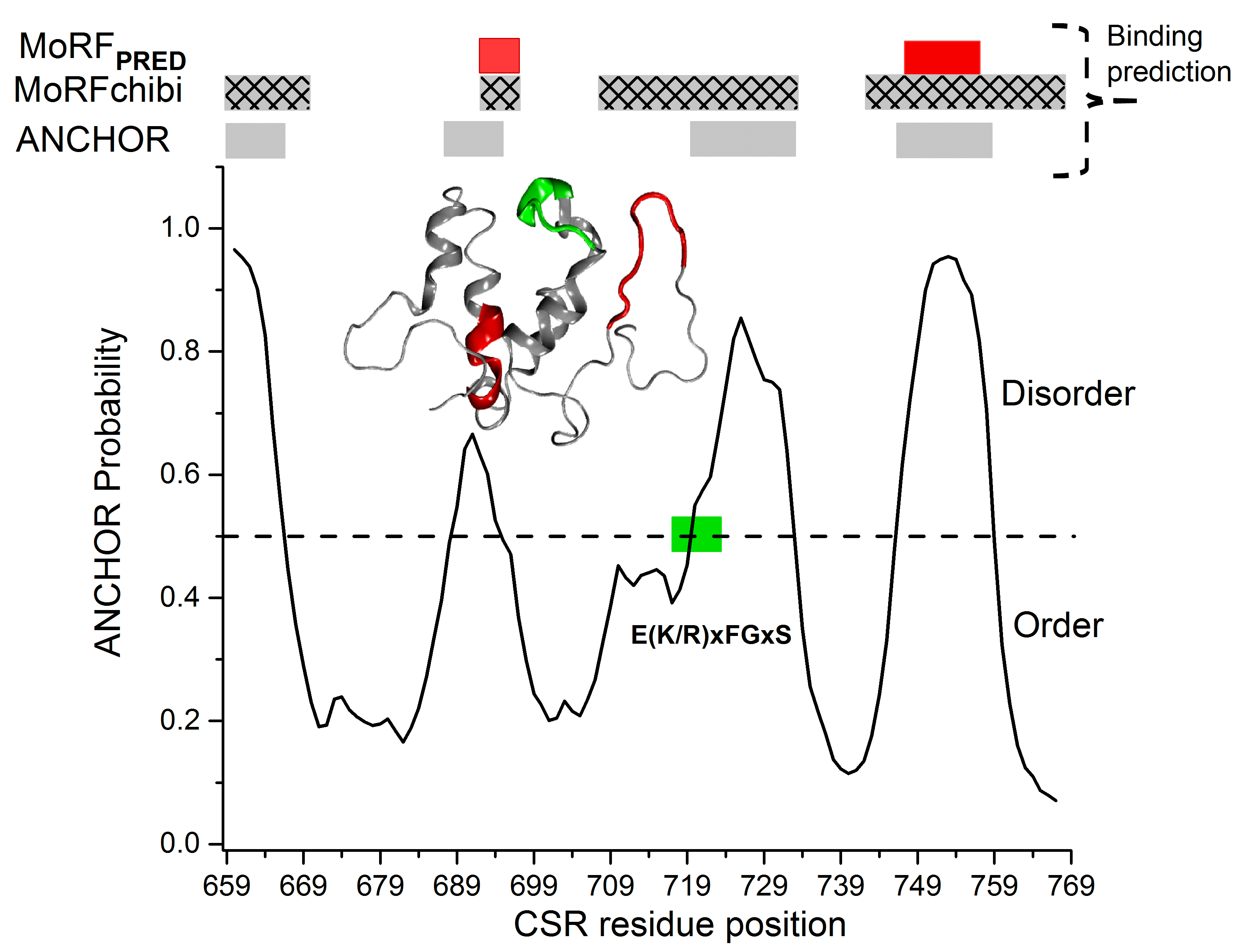


**Supplemental Fig S3.** Disorder prediction form ANCHOR probability scores for CSR region also highlighted are the binding region predictions from ANCHOR, MoRF_PRED_ and MoRFchibi. The inset structure represents CSR conformation from molecular dynamics simulations. The schematic represents x axis reveals the conserved region E(K/M)xFGxS in the CSR region of BdCESA1 is colored green and MoRF structures are colored red. One of the MoRFs with partial helical structure is refered to as alpha MoRF


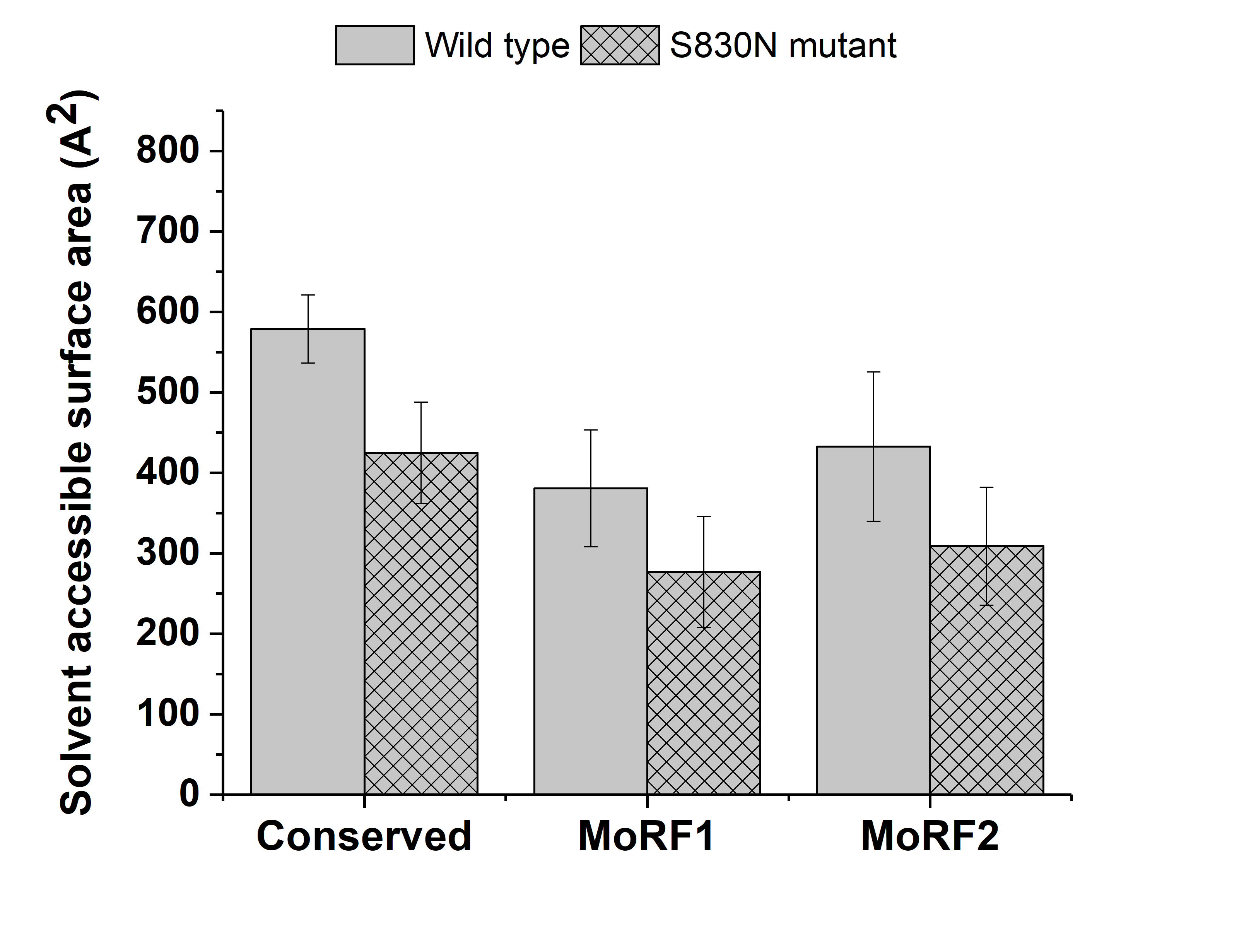


**Supplemental Fig S4.** Comparing solvent accessible surface area of conserved region, MoRF1, and MoRF2 in wild type and mutant CESA.
